# A Predictive Model for Anemia and Coronary Heart Disease Based on Bidirectional Two-Sample Mendelian Randomization and Machine Learning

**DOI:** 10.2174/0118715303427508251007080833

**Published:** 2025-10-27

**Authors:** Yan Zhang, Sheng Fan, Pengcheng Ma, Yunhong Xia, Zeping Hu

**Affiliations:** 1 Department of Cardiology, The First Affiliated Hospital of Anhui Medical University, Anhui Public Health Clinical Center, Hefei, 230022, China;; 2 Department of Oncology, The First Affiliated Hospital of Anhui Medical University, Anhui Public Health Clinical Center, Hefei, 230032, China

**Keywords:** Coronary heart disease, anemia, mendelian randomization, machine learning, biomarker, *IFIH1*, *APBB2*

## Abstract

**Introduction:**

Anemia has been linked to an increased risk of coronary heart disease (CHD), yet the underlying causal relationship remains unclear. This study aimed to investigate the bidirectional associations between anemia and CHD using a multi-method approach.

**Methods:**

Data were obtained from the European FinnGen biobank and the Gene Expression Omnibus (GEO) database. Mendelian Randomization (MR) analysis was performed with instrumental variables (IVs). The study assessed causal robustness using MR methods and sensitivity analysis, followed by differential expression analysis and weighted gene co-expression network analysis (WGCNA) to screen for the core genes. Further, machine learning algorithms, such as least absolute shrinkage and selection operator (LASSO), random forest (RF), and support vector machine (SVM) algorithms, were applied to screen for key diagnostic genes. Additionally, the CIBERSORT algorithm was used to analyze immune cell infiltration, and validation was conducted using an *in vitro* oxidized low-density lipoprotein (ox-LDL)-induced endothelial cell model and western blot experiments.

**Results:**

MR analysis revealed a positive causal link among vitamin B12 deficiency anemia, hemolytic anemia, and coronary heart disease, while cardiovascular events appeared to have a negative association with hemolytic anemia. Integrated bioinformatics analysis identified six core genes involved in immune response, inflammation, and lipid metabolism. To improve the accuracy of key gene screening and avoid bias from a single method, this study combined multiple machine learning algorithms for comprehensive analysis, ultimately identifying *IFIH1* and *APBB2* as potentially valuable diagnostic biomarkers, and revealing affected macrophages, mast cells, and T cells infiltration. *In vitro* experiments confirmed altered expression of *IFIH1* and *APBB2* upon ox-LDL treatment, supporting their role in CHD pathogenesis.

**Discussion:**

The findings of this study suggest that vitamin B12 deficiency anemia and hemolytic anemia are potential risk factors for CHD. The identification of IFIH1 and APBB2 as key biomarkers provides novel insights into the molecular mechanisms underlying CHD development in the context of anemia, offering potential targets for early diagnosis and personalized intervention strategies.

**Conclusion:**

This study, through the integration of MR, transcriptomics, and machine learning methods, has for the first time revealed the causal role of vitamin B12 deficiency anemia and hemolytic anemia in the occurrence of CHD, and identified *IFIH1* and *APBB2* as potential biomarkers. This research study has provided a new theoretical basis and research direction for understanding the molecular link between anemia and CHD and for improving clinical early warning systems.

## 1 INTRODUCTION

Cardiovascular events remain a leading global health burden. Notably, coronary heart disease (CHD), also known as ischemic heart disease, is the most prevalent and a major cause of mortality worldwide [1-3]. Data from 2021 across 204 countries indicated that CHD accounts for approximately 9 million deaths annually [4]. While traditional risk factors, such as age, hypertension, hyperlipidemia, diabetes, obesity, and smoking, are well-established, growing observational evidence suggests that anemia is also linked to an increased risk of CHD.

The World Health Organization defines anemia as hemoglobin levels below 13 g/dL in men and 12 g/dL in women. It is a major global public health issue, affecting nearly one-third of the population [5]. In addition, anemia is prevalent in individuals with CHD, impacting approximately 10% to 20% of patients [6], and is recognized as a risk factor for CHD [7]. A historical cohort study found anemia to be associated with an increased incidence of CHD [8]. Furthermore, several studies have indicated that anemia is linked to reduced survival rates in patients with acute coronary syndrome [9, 10]. However, traditional observational studies struggle to establish a clear causal relationship between anemia and CHD due to potential confounding factors, including BMI, age, and sex.

Mendelian randomization (MR) is a statistical method used in modern epidemiology to infer causal relationships [11]. It leverages genetic variants linked to exposure factors as instrumental variables (IVs) to assess the causal effect of an exposure on an outcome [12]. During gamete formation, genetic alleles are randomly assigned, resembling a natural randomized controlled trial and reducing confounding bias [13]. Moreover, as genetic variants are fixed at conception, MR minimizes the risk of reverse causation, enhancing the reliability of causal inference [14]. Most existing studies on anemia and CHD have focused on total anemia or iron-deficiency anemia, without distinguishing between different etiologies. This research study aimed to investigate the causal correlations between various types of anemia and CHD using MR analysis, followed by machine learning to identify key gene features and develop predictive models for CHD diagnosis.

## 2 MATERIALS AND METHODS

### 2.1 Study Design

We used the International Classification of Diseases (ICD) codes to define anemia and CHD in the datasets, followed by a two-sample, bidirectional MR analysis to explore their causal relationship. The primary results were evaluated using inverse-variance weighted (IVW), MR-Egger, and weighted median methods. Sensitivity analyses, including MR-Egger intercept, leave-one-out, and Mendelian randomization pleiotropy residual sum and outlier (MR-PRESSO) tests, were performed to assess the stability and reliability of results. Significant single-nucleotide polymorphisms (SNPs) from MR findings were mapped to corresponding genes. Based on the datasets downloaded from the GEO database, we conducted differential expression analysis and weighted gene co-expression network analysis (WGCNA) to identify core genes associated with both conditions. Subsequently, feature gene selection was conducted by employing the least absolute shrinkage and selection operator (LASSO), random forest (RF), and support vector machine (SVM) algorithms. We examined the role of the key genes (*IFIH1* and *APBB2*) *via* receiver operating characteristic (ROC) curve analysis, as well as their underlying function in an endothelial cell injury model established by using oxidized low-density lipoprotein (ox-LDL).

### 2.2 Data Sources

The data on anemia and CHD were collected from the European FinnGen biobank (https://www.finngen.fi/en) (Table **1**). To reduce potential population stratification, both exposure and outcome cohorts were restricted to participants of European ancestry, with additional cases of anemia and CHD excluded. All studies within the FinnGen program have been approved by local institutional review boards and ethics committees. For gene expression analysis, CHD-related microarray datasets were retrieved from the GEO database. The dataset GSE100927 was used as the training set, containing 104 samples (35 healthy controls and 69 CHD cases), while GSE66360 served as the validation set, comprising 99 samples (50 controls and 49 CHD cases).

### 2.3 Selection of IVs

**Fig. (1):**
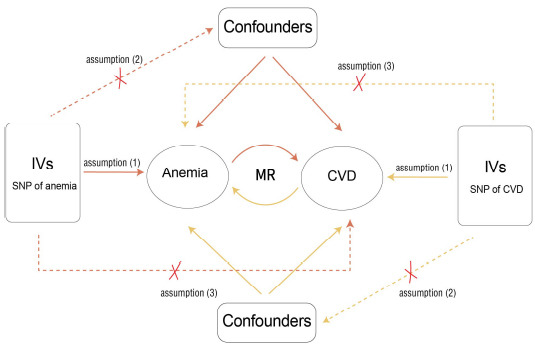
Assumptions and study design flowchart of the Mendelian randomization (MR) study.

In MR studies, the selection of IVs follows three core assumptions (Fig. **1**): (1) relevance: SNPs are closely associated with the exposure; (2) independence: SNPs are independent of confounders; (3) exclusion restriction: SNPs affect the outcome only through the exposure [15]. The IVs selection criteria were as follows: (1) due to the limited number of genome-wide significant SNPs (*p* < 5 × 10^−8^) identified in anemia-related GWAS (less than 5), and drawing upon prior studies with similar objectives, we selected SNPs with suggestive genome-wide significance (*P* < 5 × 10^−6^) as IVs to ensure an adequate number of IVs. Sensitivity analyses were then performed to assess the stability and reliability of the results [16, 17]. (2) To ensure independence, linkage disequilibrium (LD) was removed among SNPs (r^2^ < 0.001 (window size = 10,000 kb)), selecting only independent SNPs. Population stratification can be mitigated by this method. (3) The strength of the IVs-exposure association was assessed using R^2^ (variance explained) and F-statistics. R^2^ was calculated as R^2^ = β^2^ (1 – EAF) × 2EAF, where EAF is the effect allele frequency, and the F-statistic was computed as F = R^2^ (N – K – 1) / [K (1 – R^2^)], where K is the number of SNPs and N is the GWAS sample size. An F-statistic > 10 indicates strong instruments and reduces bias from weak associations. (4) SNPs potentially associated with the outcome were manually screened and excluded using the PhenoScanner database [18].

### 2.4 MR Analysis for Determining the Underlying Causal Association Between Anemia and CHD

We applied the IVW method for the causal association assessment. As complementary approaches, the MR-Egger, weighted median, and simple median methods were further used for the causal relationship verification. The weighted median method provides a consistent estimate as long as at least 50% of the weight comes from valid IVs. MR-Egger regression not only estimates causal effects, but also detects potential directional pleiotropy through its intercept term [19]. The MR-PRESSO method was used to identify and correct horizontal pleiotropy and outliers to reduce pleiotropy [20]. Sensitivity analyses included assessment of horizontal pleiotropy using the MR-Egger intercept and evaluation of SNP heterogeneity *via* Cochran’s Q test; a *p*-value > 0.05 suggested no significant pleiotropy or heterogeneity. Additionally, leave-one-out analysis was performed by iteratively removing each SNP and recalculating the MR results to assess the influence of individual IVs. MR findings were visualized using the TwoSampleMR package in R.

### 2.5 WGCNA for the CHD-related Gene Module

Using the “limma” R package, differentially expressed genes (DEGs) in the CHD and normal samples were identified based on *p* < 0.05 and |log_2_FC| > 0.585. Subsequently, based on CHD sample expression profiles, a gene co-expression network was constructed by applying the WGCNA package. Further analysis was performed using genes with a standard deviation greater than 0.5 [21]. The gene expression matrix was then converted into the corresponding adjacency matrix, and gene modules were identified using a topological overlap clustering method. By calculating module significance (MS) and gene significance (GS), the associations between gene modules and clinical traits were evaluated [22].

### 2.6 Potential Candidate Genes Identified by Functional Enrichment Analysis

These core genes were subjected to Gene Ontology (GO) and Kyoto Encyclopedia of Genes and Genomes (KEGG) functional enrichment analyses utilizing the “clusterProfiler” and “enrichplot” R packages (terms with *p*<0.05 and q<0.05 were selected) [23]. The “circlize” R package was used to visualize the chromosomal locations of these core genes.

### 2.7 Overlapping Genes for the Candidate Diagnostic Markers

Next, we analyzed the MR results, filtering IVs with IVW *p* < 0.05 and consistent odds ratios (OR) across five methods, and mapped these IVs to their corresponding genes. The disease-associated modules from WGCNA that had a core module *p* < 0.05 were identified. Using the “VennDiagram” package, we determined the intersection of differentially expressed genes, WGCNA core module genes, and MR-identified genes, which were defined as core genes [24]. To identify the final core genes, these candidate diagnostic biomarkers were selected through a screening process employing LASSO, RF, and SVM algorithms. These analyses were performed using the 'glmnet', 'randomForest', and 'svmRadial' R packages, respectively, with hyperparameter optimization achieved *via* 10-fold cross-validation [25-27]. The diagnostic efficiency of these diagnostic markers in CHD samples was analyzed using the “pROC” R package, and a nomogram model was developed using the “rms” package. Calibration curves were generated using the “regplot” package for the model accuracy, and decision curves were plotted using the “ggDCA” R package for the clinical utility analysis.

### 2.8 Analysis of Immune Infiltration

We calculated the immune infiltration score of 22 immune cell types to evaluate their difference in control and CHD samples using the “CIBERSORT” R package with the rank-sum test [28]. The relationships between diagnostic markers and immune infiltration score were evaluated using correlation analysis.

### 2.9 Cell Culture and Western Blotting Analysis of the Protein Expression Difference

High-glucose DMEM containing 10% FBS and 1% penicillin-streptomycin was used to culture human umbilical vein endothelial cells (HUVECs) purchased from the University of the Chinese Academy of Sciences in 5% CO_2_ at 37°C with saturated humidity. The medium was renewed every two days, and the cells were passaged every 2-3 days. Upon reaching 80%-90% confluence, HUVECs were digested with 0.25% trypsin for 3-5 minutes, centrifuged, washed, and resuspended in DMEM at a concentration of 1 × 10^6^ cells/mL. After 24 hours of culture, 1 mL of 100 μg/mL ox-LDL was added to induce cell injury for another 24 hours. For the western blotting analysis, the harvested cells were lysed using RIPA lysis buffer (Biosharp BL504A), and the protein concentration was measured using a BCA kit (Biosharp BL521A). Next, 10% SDS-PAGE was applied to separate the proteins, which were transferred onto a PVDF membrane. Then, the membrane was blocked with 5% non-fat milk and incubated overnight at 4°C with primary antibodies against IFIH1 (1:2000, 21775-1-AP) as well as housekeeping control β-actin (1:1000, MA5-15739) and APBB2 (1:300, 13177-1-AP). After washing, the membrane was further incubated with secondary antibodies (HRP-conjugated goat anti-rabbit IgG) at 37°C for 2 hours, and chemiluminescence was used for protein detection. Target band intensities were normalized to β-actin using ImageJ software.

### 2.10 Statistical Analysis

Data analysis and graphical visualization were completed using R software (4.2.3) and Sangerbox 2 auxiliary platform as well as GraphPad Prism software (8.0.2) [29]. The Wilcoxon rank-sum test and unpaired t test were used for comparing differences between groups, and the Spearman method was employed for the correlation analysis. A *p*<0.05 stood for statistical significance.

## 3 RESULTS

### 3.1 Forward MR Identified Vitamin B12 Deficiency Anemia and Hemolytic Anemia as Potential Risk Factors for CHD

**Fig. (2):**
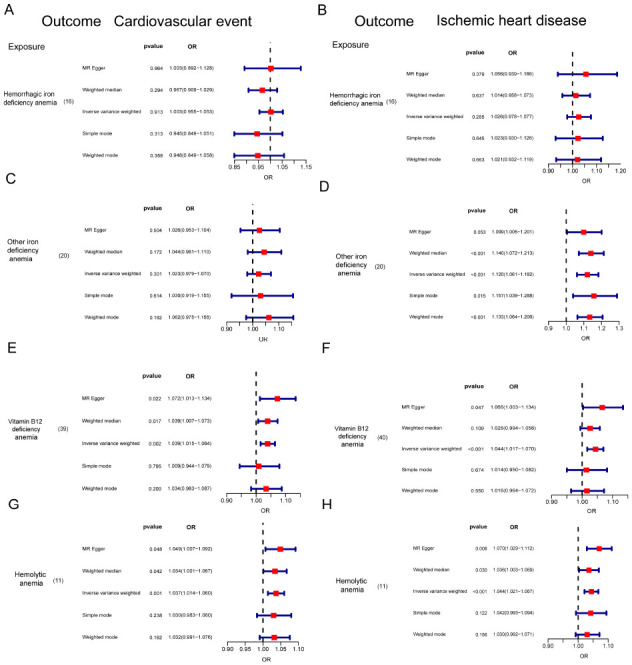
MR predicted the risk association of anemia causing CHD. (**A-D**) The impact of various anemia types on the outcome of cardiovascular events. (**E-H**) The impact of various anemia types on the outcome of ischemic heart disease. (OR>1 for the exposure increasing the risk of outcome, OR=1 for no significant correlation between the exposure and outcome, OR-<1 for the exposure decreasing the risk of outcome; 95% CI of 1 indicates the conclusion without statistical significance).

The forward MR results were visualized by the effect size plot with OR (red dot representing the OR value >1 indicated that the exposure was linked to a higher risk of the outcome, OR =1 indicated no causal relationship, OR<1 indicated that the exposure reduced the risk of the outcome), 95% confidence interval (CI, the blue lines on either side of the red square representing the 95% CI; at CI = 1, the result was not statistically significant), *p* value, and the numbers of SNPs (Figs. **2A**-**H**). IVW analysis revealed that iron deficiency anemia may increase the risk of ischemic heart disease (OR = 1.120; 95% CI: 1.061–1.182; *p* < 0.001). This finding was supported by MR-Egger analysis (OR = 1.099; 95% CI: 1.005–1.201; *p* = 0.053) (Fig. **2D**). A positive causal association between vitamin B12 deficiency anemia and cardiovascular events (OR = 1.039; 95% CI: 1.015-1.064; *p* = 0.002) was consistent with findings from MR-Egger analysis (OR = 1.072; 95% CI: 1.013-1.134; *p* = 0.022) and the weighted median method (OR = 1.039; 95% CI: 1.007-1.073; *p* = 0.017) (Fig. **2E**), and vitamin B12 deficiency anemia showed a positive causal relationship with ischemic heart disease (OR = 1.044; 95% CI: 1.017 -1.070; *p* < 0.001), being consistent with MR-Egger analysis (OR = 1.066; 95%CI: 1.003 -1. 134; *p* = 0.047) (Fig. **2F**). The hemolytic anemia increased the risk of cardiovascular events in the IVW (OR = 1.037, 95% CI: 1.014-1.060, *p* = 0.001) and MR-Egger (OR = 1.049, 95% CI: 1.007-1.092, *p* = 0.048) analyses (Fig. **2G**). In addition, the hemolytic anemia also increased the risk of ischemic heart disease, as demonstrated by IVW (OR =1.044; 95% CI: 1.021-1.067; *p* <0.001) and MR-Egger analyses (OR = 1.070; 95% CI: 1.029-1.112; *p* = 0.008) (Fig. **2H**). In contrast, no significant association was observed between iron deficiency anemia and cardiovascular events across any analytical method.

### 3.2 Reverse MR Showed that the Cardiovascular Events May Reduce the Risk of Hemolytic Anemia

**Fig. (3):**
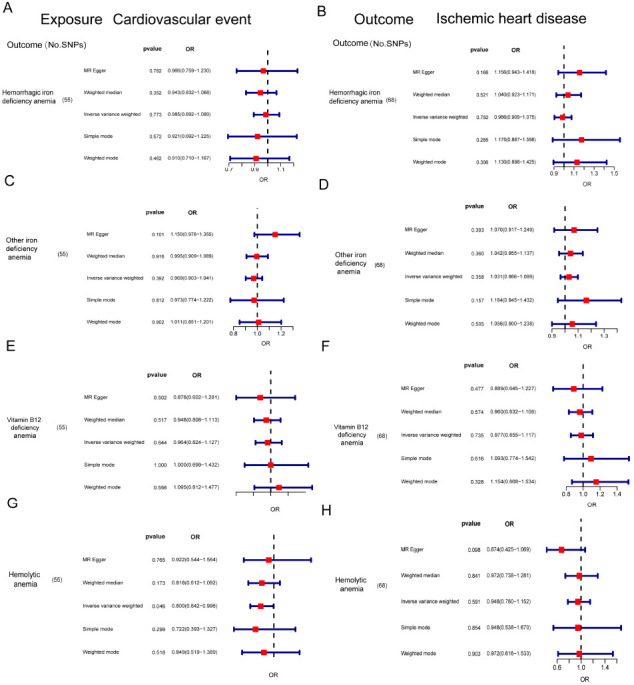
MR predicted the risk association of CHD causing anemia. (**A-D**) The impact of cardiovascular events on the outcome of various anemia types. (**E-H**) The impact of ischemic heart disease on the outcome of various anemia types. (OR>1 for the exposure increasing the risk of outcome, OR=1 for no significant correlation between the exposure and outcome, OR-<1 for the exposure decreasing the risk of outcome; 95% CI of 1 indicates the conclusion without statistical significance).

Reverse MR analysis (Figs. **3A-H**) suggested that cardiovascular events may be inversely associated with hemolytic anemia, with IVW (OR = 0.800; 95% CI: 0.642-0.996; *p* = 0.046) analysis supporting it (Fig. **3G**). Other cardiovascular events, ischemic heart disease, and anemia showed no significant causal relationships.

### 3.3 Heterogeneity and Sensitivity Analysis

**Fig. (4):**
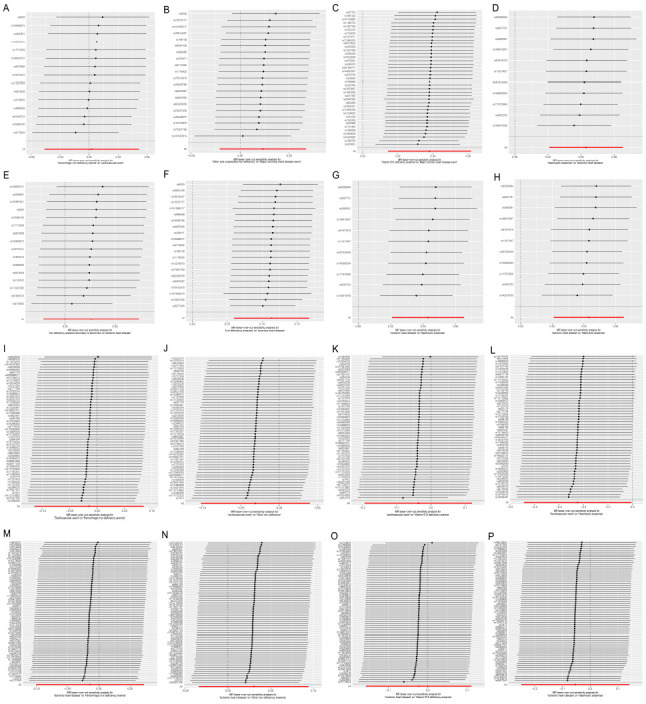
Heterogeneity and sensitivity analyses. (**A-P**) Leave-one-out method for heterogeneity and sensitivity analyses.

Heterogeneity among IVs in MR analysis was assessed by Cochran's Q test, and a *p*-value < 0.05 indicated significant heterogeneity, meaning the IVs yielded inconsistent effect estimates. The MR-Egger regression intercept was employed for the pleiotropy analysis of robustness and reliability, with the intercept being close to zero and *p* > 0.05 indicating no horizontal pleiotropy. Our results showed cardiovascular events to be the outcome; vitamin B12 deficiency anemia showed significant heterogeneity (Q = 62.516, *p* = 0.007), but no evidence of horizontal pleiotropy was observed (Table **2**). When ischemic heart disease was the outcome, other types of iron deficiency anemia and vitamin B12 deficiency anemia showed significant heterogeneity (Q = 36.305, *p* = 0.014), yet there was still no evidence of significant pleiotropy (Table **2**). When cardiovascular events or ischemic heart disease were the exposure, vitamin B12 deficiency anemia and other iron deficiency anemia showed significant heterogeneity (*p*<0.05), while the hemolytic anemia had no significant heterogeneity or pleiotropy (*p*>0.05) (Table **2**), indicating more robust causal effects. Overall, most anemia subtypes met instrumental variable assumptions, though results for other iron deficiency anemia should be interpreted cautiously due to possible confounding factors. Furthermore, a leave-one-out sensitivity analysis was performed to determine whether any outlier instrumental variables (IVs) had a significant impact on the overall results. The analysis showed that most SNPs had effect estimates close to zero after removal, with confidence intervals overlapping the overall effect estimate, indicating minimal influence on the final results (Figs. **4A-P**) and suggesting the results to be fairly robust.

### 3.4 Core genes Involved in Immune Regulation and Lipid Metabolism

**Fig. (5):**
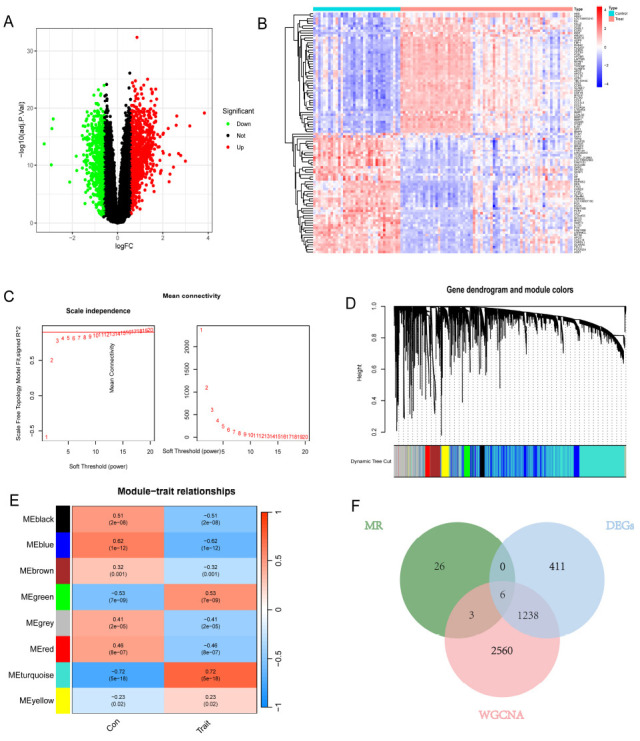
WGCNA for the CHD-related gene module. (**A**) Volcano plot for the differential expression analysis. (**B**) Heatmap of the expressions of the top 25 DEGs. (**C**) Soft threshold selection for the scale-free network. (**D**) Gene dendrogram and module colors plot. (**E**) Correlation heatmap of the gene module and traits. (**F**) Venn plot for the overlapping genes.

**Fig. (6):**
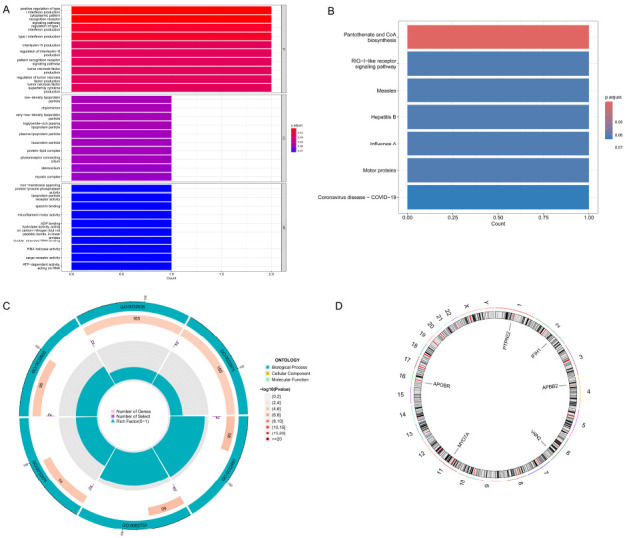
Analysis of gene functions and characteristics. (**A, B**) GO and KEGG enrichment analyses of six core genes. (**C, D**) The position analysis of six core genes.

Subsequently, the differential expression analysis identified 1,655 DEGs, including 667 downregulated DEGs and 988 upregulated DEGs (Fig. **5A**); the expressions of the top 25 DEGs are shown in Fig. (**5B**). We applied WGCNA to explore CHD-related gene modules. When the soft threshold was set to 6, the network exhibited scale-free properties (Fig. **5C**). After modules merged with correlation > 0.25 (Fig. **5D**), a total of 8 modules (turquoise, yellow, brown, black, blue, green, gray, and red) were identified using dynamic tree cutting, and the turquoise and blue modules were significantly correlated with the CHD trait (Fig. **5E**). By intersecting 35 MR-identified CHD-related genes, 1655 DEGs, and key module genes, we obtained 6 overlapping genes as core genes (Fig. **5F**). We examined the function of these genes in the GO and KEGG databases. These genes were closely linked to the immune response (regulation of type I interferon production, positive regulation of type I interferon production, pattern recognition receptor signaling pathway), lipoprotein metabolism (low-density lipoprotein particle, chylomicron, very-low-density lipoprotein particle), and inflammation (interleukin-6 production, tumor necrosis factor production) (Fig. **6A**). Meanwhile, KEGG analysis showed these genes to be enriched in pantothenate and CoA biosynthesis pathways (Fig. **6B**). To further explore the chromosomal organization and inheritance patterns of the core genes, we constructed a circos plot to visualize their genomic locations (Figs. **6C** and **D**); the circos plot showed the chromosomal distribution of core genes, highlighting their locations: *APOBR* on chromosome 16, *MYO7A* on 12, *PTPN22* on 1, *IFIH1* on 2, *APBB2* on 3, and *VNN2* on 6.

### 3.5 APBB2 and IFIH1 Emerged as Key Diagnostic Biomarkers for CHD

**Fig. (7):**
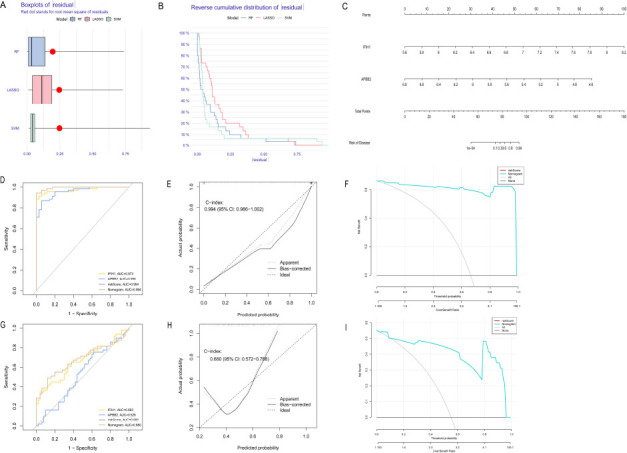
Identifying the biomarkers and developing a nomogram model. (**A**) The absolute values of residuals (|residual|) for RF, LASSO, and SVM models. (**B**) The reverse cumulative distribution plot illustrates the cumulative distribution of the absolute values of residuals (|residual|) across different models. (**C**) A developing nomogram model. (**D**) ROC analysis for the classifier efficiency evaluation in the training set. (**E**) Calibration curve for the predicting accuracy assessment of the nomogram model. (**F**) Decision curve for the clinical application value evaluation. (**G-I**) ROC analysis (**G**), calibration curve (**H**), and decision curve (**I**) in the validation sets.

Next, we further selected the potential candidate diagnostic genes using three machine learning algorithms, RF, LASSO, and SVM; *APBB2* and *IFIH1* were consistently ranked among the top predictors and selected as candidate diagnostic markers. The reverse cumulative distribution of absolute residuals (|residual|) illustrated model error concentration, and a steeper descent indicated more predictions near true values. The boxplots of residuals (|residual|) showed these two genes in the SVM model to have the most concentrated residual distribution and the lowest RMS (Fig. **7A**), indicating the highest prediction accuracy. The reverse cumulative distribution of the absolute residuals (|residual|) showed that the SVM model’s values decreased most rapidly, particularly in the region of smaller residuals (Fig. **7B**), indicating most predicted values to closely match the actual values. To quantify the risk probability of patients, a nomogram model was developed by combining the two diagnostic markers (Fig. **7C**). We examined the diagnostic efficiency of markers and the nomogram model, and the ROC analysis showed the AUC value for *APBB2*, *IFIH1*, and the nomogram model to be 0.958, 0.973, and 0.994, respectively (Fig. **7D**). The calibration curve showed excellent classifier ability (C-index = 0.994, 95%CI: 0.986-1.002), and the calibration curve closely aligned with the ideal curve (Fig. **7E**), indicating the predicted probabilities to be highly consistent with the actual probabilities. The decision curve indicated the high net benefit of the nomogram model (Fig. **7F**), suggesting the good clinical application value of our model. In addition, similar results were observed in the verification set; ROC revealed the good diagnostic efficiency of markers and nomogram model (Fig. **7G**), and the calibration curve (C-index=0.680, 95%CI: 0.572–0.788) and decision curve suggested the excellent predictive performance and clinical application value of our model (Figs. **7H** and **I**).

### 3.6 Immune Cell Infiltration Relationship of IFIH1 and APBB2 and ox-LDL-induced Modulation in CHD

**Fig. (8):**
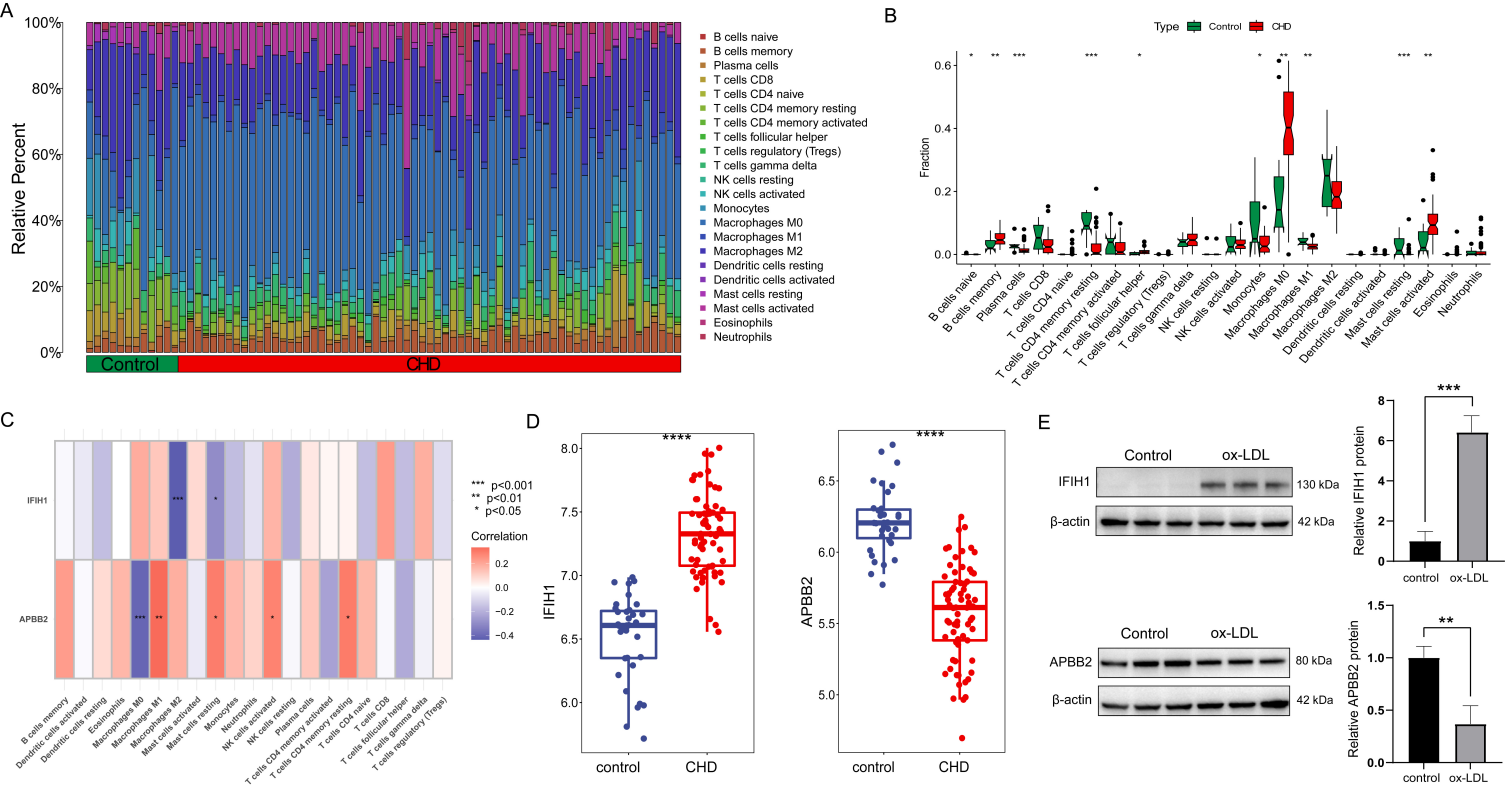
The correlation between biomarkers and immune cell infiltration. (**A**) The proportion of various immune cell types in different samples. (**B**) The immune infiltration differences among various immune cell types in different samples. (**C**) The correlation between the biomarkers and the immune cell infiltration score. (**D**) The biomarker expression difference in different samples. (**E**) Western blotting analysis for the protein expression difference in different treatments (*p<0.05, **p<0.01, ***p<0.001, ****p<0.0001).

The association between these two biomarkers and immune cell infiltration was analyzed. First, CIBERSORT analysis revealed the proportion changes of immune cell composition in CHD patients, including a decrease in naive B cells and naive CD4^+^ T cells, and an increase in memory B cells, regulatory T cells, and activated memory CD4+ T cells in the CHD group (Fig. **8A**), suggesting a possible enhancement of humoral and regulatory immune responses. Compared to the control group, CHD patients exhibited notable changes in immune cell composition, including a decrease in naive B cells and CD8^+^ T cells, an increase in activated memory CD4^+^ T cells and regulatory T cells, elevated levels of plasma cells and multiple macrophage subtypes (especially M0 and M2), as well as activation of mast cells (Fig. **8B**). These alterations may reflect the complex immune responses and inflammatory state present in CHD patients. Correlation analysis demonstrated the *IFIH1* expression to be mainly negatively related to resting mast cells and M2 macrophages, while the *APBB2* genes were significantly positively correlated with resting mast cells, resting memory CD4^+^ T cells, activated NK cells, and M1 macrophages (Fig. **8C**). These correlations suggested that *IFIH1* and *APBB2* may work together to regulate specific immune cell functions and influence inflammatory responses. It is worth noting that these associations provided clues for further research, but they still need to be verified by functional experiments or longitudinal studies.

Finally, we examined the expressions of *IFIH1* and *APBB2* in the training set and found *IFIH1* to be significantly up-regulated, while *APBB2* was significantly down-regulated in the CHD samples (Fig. **8D**, *p*<0.0001). Additionally, we detected the protein expression difference of two biomarkers in *in vitro* cells with ox-LDL treatment; the results presented the IFIH1 protein to be increased in the ox-LDL treatment group, whereas the APBB2 protein in the ox-LDL treatment group was reduced (Fig. **8E**).

## 4 DISCUSSION

CHD and anemia represent two major global health burdens, with emerging evidence suggesting a complex interplay between them. This research study conducted a two-sample bidirectional MR analysis to explore the causal relationships between different types of anemia and CHD phenotypes. Our bidirectional MR analysis identified a positive causal effect of vitamin B12 deficiency anemia and hemolytic anemia on CHD risk. However, reverse MR suggested that cardiovascular events may be inversely associated with hemolytic anemia, implying a complex interplay. No other types of anemia demonstrated significant causal links with CHD.

In patients with CHD, anemia is a common comorbidity that affects approximately 15% to 61% of hospitalized cardiovascular patients globally [30]. Among these, malnutrition-related anemia is most common (47.1%), with renal anemia (23.5%), iron deficiency anemia (25.5%), and megaloblastic anemia (21.6%) being the most prevalent subtypes [31], highlighting anemia as a notable risk factor for CHD [32]. Previous research suggested that CHD may contribute to anemia through several indirect mechanisms: CHD patients often have concurrent kidney dysfunction, leading to loss of recombinant human erythropoietin (EPO), iron, transferrin, and others through urine. The activation of the renin-angiotensin-aldosterone system (RAAS) can impair kidney function and reduce EPO production. Long-term aspirin use in CHD patients may cause gastrointestinal bleeding, contributing to anemia [33, 34]. Notably, our MR analysis indicated that cardiovascular events may reduce the risk of hemolytic anemia (OR = 0.8, *p* = 0.046), pointing to a potential protective association. Several mechanisms could underlie this observation. First, patients with CHD typically receive comprehensive medical management, including lipid-lowering, anti-inflammatory, and immunomodulatory therapies, which may stabilize red blood cell membranes and decrease hemolysis. Second, CHD-associated alterations in immune regulation might suppress excessive complement activation or macrophage-driven erythrocyte clearance, thereby lowering the risk of hemolysis. Third, adaptive erythropoietic responses triggered by chronic cardiovascular stress may enhance red blood cell turnover and compensate for hemolytic loss. These hypotheses highlight that CHD may exert differential effects on anemia subtypes, increasing the risk of some while potentially attenuating hemolytic anemia.

However, the relationship between anemia and CHD is more complex. Some studies have suggested anemia to significantly increase CHD risk [35], while research on individuals with vascular disease indicates that higher hemoglobin levels may reduce atherosclerosis severity [36], implying that anemia might genuinely promote the onset of CHD through certain pathways. Consistent with this notion, our results highlighted vitamin B12 deficiency anemia and hemolytic anemia to be positively associated with CHD occurrence. One possible explanation is that these two subtypes are more directly linked to inflammation, immune dysregulation, and metabolic disturbances. This is further supported by relatively larger sample sizes and stronger genetic instruments in the datasets, enhancing the robustness of the causal inference. By contrast, other anemia subtypes, such as iron deficiency anemia, may have limited case numbers or weaker genetic signals, reducing statistical power and resulting in less stable associations. To enhance the clinical application value of our research study, we applied three machine learning methods to identify two crucial diagnostic markers based on the CHD-related signature. Only the IVs of vitamin B12 deficiency anemia overlapped with both DEGs and module genes, leading to the identification of six core genes. Functional enrichment analysis further indicated that these genes are implicated in a wide range of biological processes, including inflammation, lipid metabolism, immune response, and cellular functions, underscoring their multifaceted roles in maintaining homeostasis and mediating disease-related pathways.

To improve the specificity and clinical relevance of the six core genes identified, we applied LASSO, RF, and SVM algorithms for further selection. After screening with all three algorithms, two characteristic genes, *IFIH1* and *APBB2*, were consistently selected. CHD is primarily driven by atherosclerosis, a chronic progressive inflammatory vascular disease [37, 38]. The *IFIH1* gene, which encodes melanoma differentiation-associated protein 5 (MDA5), plays a crucial role as an innate immune regulator, specifically in the early detection of viral infections [39]. Amyloid beta A4 precursor protein-binding family B member 2 (*APBB2*) is linked to the increasing plasma beta-amyloids [40], suggesting that they may exert indirect effects on the onset and progression of the disease through regulating inflammatory responses and vascular wall structure. The relationship between these genes and immune cell infiltration in CHD across 22 immune cell types was analyzed [41]. The results showed *IFIH1* to be negatively linked to mast cells and M2 macrophages. *APBB2* showed negative correlations with M0 macrophages and positive correlations with NK cells, T cells, M1 macrophages, and mast cells. Previous studies have shown macrophage polarization to play a key role in AS progression. The balance between pro-inflammatory M1 and anti-inflammatory M2 macrophages influences plaque stability [42, 43]. Mast cells, abundant in AS plaques, contribute to vascular dysfunction, extracellular matrix degradation, and immune cell recruitment, promoting inflammation and plaque rupture [44]. T cells are also extensively involved in AS formation [45]. These findings suggest that *IFIH1* and *APBB2* are closely linked to the inflammatory processes in CHD, potentially influencing disease progression through modulation of macrophage phenotypes and mast cell activation.

Furthermore, we constructed a nomogram model and examined its clinical diagnostic value; the results presented the good diagnostic efficiency of our model and biomarkers. In *in vitro* assay, the high-fat treatment increased the expression of IFIH1 but reduced the expression of APBB2 in HUVECs, further confirming their involvement in CHD pathogenesis. The *APBB2* belongs to the Fe65 family of scaffold proteins, which includes APBB1 (Fe65) and APBB3 (Fe65L2) [46]. Studies have shown APBB2 to enhance APP formation and reduce apoptosis sensitivity [47]. It interacts with the very low-density lipoprotein receptor (VLDLR) family and apolipoprotein E (ApoE), which are well-established regulators of lipid homeostasis and endothelial function [48], suggesting its role in modulating CHD processes. Given the well-established links among ApoE, VLDLR, and CHD progression, these findings suggest that APBB2 may contribute to CHD development through pathways involving lipid metabolism, energy homeostasis, and cell cycle regulation. Overexpression of *IFIH1* can independently trigger IFN-β production, and upon activation, it acts as an adaptor molecule that stimulates macrophages to produce type I interferons [49], thereby initiating immune and inflammatory responses. *IFIH1* has been considered a shared genetic risk factor for both autoimmune diseases and coronary artery disease [50]. Genetic variants in *IFIH1* are linked to conditions, such as rheumatoid arthritis, type 1 diabetes, vitiligo, multiple sclerosis, psoriasis, and lupus, largely due to its role in chronic type I interferon activation, which drives autoinflammatory and autoimmune processes [51]. Given that atherosclerosis, the underlying pathology of CHD, is also a chronic inflammatory disease, the findings have suggested a potential mechanistic link between gene *IFIH1* and CHD.

This work has included several limitations. First, this study primarily relied on GWAS and GEO database data from European populations, which are genetically homogeneous; however, the number of genome-wide significant SNPs available in anemia-related GWAS is limited. This may result in insufficient population generalizability and weak instrumental variable bias. In the future, we will incorporate multi-ethnic large-scale cohorts and, as more GWAS data accumulate, use strict threshold-based screening of instrumental variables to enhance the robustness and generalizability of causal inference. Second, despite employing multiple MR methods and sensitivity analyses, it remains challenging to completely rule out potential multivariable effects or confounding biases. Additionally, immune infiltration analysis can only reveal correlations and lacks direct causal evidence. To address this aspect, we will further combine multivariate MR, colocalization analysis, single-cell sequencing, and animal model experiments to validate the true causal relationship between key genes and immune cells, and further elucidate the underlying mechanisms. Finally, this study's *in vitro* experiments only used an ox-LDL-induced endothelial injury model, which cannot fully simulate the complex pathological processes *in vivo*. Although the predictive model performed well in the training and validation sets, it still lacks large-scale prospective clinical validation. Therefore, in the future, we will combine animal models, clinical samples, and multicenter cohort studies to verify the role of key biomarkers and the stability of the predictive model, and compare and evaluate it with existing clinical scoring tools.

## CONCLUSION

In summary, this study integrated Mendelian randomization, bioinformatics analysis, and machine learning to explore the bidirectional relationship between anemia and CHD. We identified *IFIH1* and *APBB2* as key genes associated with CHD development in the context of vitamin B12 deficiency anemia. A predictive model was developed and preliminarily validated through cellular experiments, providing a foundation for future studies on the interplay between anemia and CHD. It is worth noting that the clinical applicability of these results still needs to be verified in larger populations in the future.

## Figures and Tables

**Table 1:** Overview of data sources related to phenotypes.

**Traits**	**ICD**	**Sample Size (cases/controls)**	**Population**
Cardiovascular event	I20.0 I21 I22	46959/365222	European
Ischemic heart disease	I24 I25	51589/343079	European
Hemorrhagic iron deficiency anemia	D50.0	5343/393684	European
Other iron deficiency anemia	D50.1D50.8D50.9	11336/393684	European
Vitamin B12 deficiency anemia	D51	3694/393684	European
Hemolytic anemia	D55-59	911/411270	European

**Table 2:** Heterogeneity and sensitivity analysis.

**Exposure**	**Outcome**	**Cochran’s Q Test**	**Q_*p* val**	**MR-Egger Intercept**	***p-*value**
Hemorrhagic iron deficiency anemia	Cardiovascular event	18.269	0.195	-0.001	0.990
Other iron deficiency anemia	-	20.009	0.394	-0.001	0.940
Vitamin B12 deficiency anemia	-	62.516	0.007	-0.006	0.246
Folate deficiency anemia	-	53.131	0.042	-0.004	0.412
Hemolytic anemia	-	9.629	0.474	-0.006	0.534
Hemorrhagic iron deficiency anemia	Ischemic heart disease	22.678	0.091	-0.005	0.613
Other iron deficiency anemia	-	36.305	0.014	0.003	0.598
Vitamin B12 deficiency anemia	-	46.990	0.104	0.002	0.672
Folate deficiency anemia	-	59.645	0.011	0.002	0.677
Hemolytic anemia	-	11.424	0.325	-0.012	0.177
cardiovascular event	Hemorrhagic iron deficiency anemia	76.190	0.025	0.002	0.862
-	Other iron deficiency anemia	80.412	0.011	-0.015	0.029
-	Vitamin B12 deficiency anemia	130.381	0.001	0.008	0.596
-	Folate deficiency anemia	39.656	0.928	-0.017	0.672
-	Hemolytic anemia	64.139	0.163	-0.012	0.562
Ischemic heart disease	Hemorrhagic iron deficiency anemia	80.787	0.120	-0.013	0.097
-	Other iron deficiency anemia	93.484	0.018	-0.003	0.600
-	Vitamin B12 deficiency anemia	134.363	0.001	0.008	0.529
-	Folate deficiency anemia	53.193	0.890	-0.017	0.632
-	Hemolytic anemia	71.752	0.323	0.028	0.115

## Data Availability

The datasets generated and/or analyzed during the current study are available in the GSE100927 repository (https://www.ncbi.nlm.nih.gov/geo/query/acc.cgi?acc=GSE100927) and the GSE66360 repository (https://www.ncbi.nlm.nih.gov/geo/query/acc.cgi?acc=GSE66360).

## LIST OF ABBREVIATIONS

APBB2: Amyloid Beta Precursor Protein Binding Family B Member 2
ApoE: Apolipoprotein E
AUC: Area Under The Curve
CHD: Coronary Heart Disease
CI: Confidence Interval
CIBERSORT: Cell-Type Identification By Estimating Relative Subsets Of RNA Transcripts
DEGs: Differentially Expressed Genes
EPO: Erythropoietin
GEO: Gene Expression Omnibus
GO: Gene Ontology
GS: Gene Significance
HUVECs: Human Umbilical Vein Endothelial Cells
ICD: International Classification Of Diseases
IFIH1: Interferon Induced With Helicase C Domain 1
IVs: Instrumental Variables
IVW: Inverse-Variance Weighted
KEGG: Kyoto Encyclopedia Of Genes And Genomes
LASSO: Least Absolute Shrinkage And Selection Operator
LD: Linkage Disequilibrium
MR: Mendelian Randomization
MR-PRESSO: Mendelian Randomization Pleiotropy Residual Sum And Outlier
MS: Module Significance
OR: Odds Ratios
Ox-LDL: Oxidized Low-Density Lipoprotein
RAAS: Renin-Angiotensin-Aldosterone System
RFE: Recursive Feature Elimination
RF: Random Forest
ROC: Receiver Operating Characteristic
SNPs: Single-Nucleotide Polymorphisms
SVM: Support Vector Machine
VLDLR: Very Low-Density Lipoprotein Receptor
WGCNA: Weighted Gene Co-Expression Network Analysis

## STANDARDS OF REPORTING

Strobe-MR guidelines were followed.
